# Pollinator Visitation Within a Single Crown of the Rare Rainforest Tree *Firmiana daweishanensis* Is Associated With Floral Colour Change

**DOI:** 10.1002/ece3.74490

**Published:** 2026-10-12

**Authors:** Bo‐Qiang Wang, Si‐Yue Xiao, Cheng‐Jie Li, Yan‐Mei Ping, Jin‐Guo Zhang, Wei‐Bang Sun, Jing Yang

**Affiliations:** ^1^ Yunnan Key Laboratory for Integrative Conservation of Plant Species With Extremely Small Populations, Kunming Institute of Botany Chinese Academy of Sciences Kunming China; ^2^ University of Chinese Academy of Sciences Beijing China; ^3^ Forestry and Grassland Bureau of Hekou County Hekou Yunnan China; ^4^ Maguan Gulinqing Provincial Nature Reserve Management and Protection Bureau Maguan Yunnan China; ^5^ State Key Laboratory of Plant Diversity and Specialty Crops, Kunming Institute of Botany Chinese Academy of Sciences Kunming China

**Keywords:** floral colour change, multimodal floral signals, plant species with extremely small populations, pollinator preference, rainforest canopy

## Abstract

Floral colour change can signal floral state, but its role in rainforest canopy trees remains poorly understood. We investigated colour change and pollinator visitation in *Firmiana daweishanensis*, a Plant Species with Extremely Small Populations endemic to southeastern Yunnan, China. Using a 25‐m scaffold, we observed 16 flowering branches on one adult tree for 128 branch‐hours. We combined these observations with measurements of linalool emission, nectar accumulation, reproductive function, calyx reflectance and bee visual modelling. Six hymenopteran taxa visited the tree, with *Megachile* bees dominating visitation. Mixed models showed that yellow flowers received more visits and greater cumulative visitation time per flower‐hour than red flowers within both sexes. Neither the omnibus sex tests nor the colour × sex interactions were significant. Branch‐level floral abundance and colour composition were not significantly associated with visitation. This contrast was consistent with, but did not demonstrate, selection at a fine spatial scale; limited statistical power remained an alternative explanation. Yellow flowers showed higher linalool emission and stronger modelled green‐receptor contrast against a green background. Nectar availability and reproductive function were generally greater during earlier floral stages. Neither pollinator‐excluded nor artificially geitonogamously self‐pollinated flowers produced fruit, whereas naturally exposed flowers showed 6.19% fruit set. These observations support a contribution of floral visitors to fruit set, but the absence of a hand‐cross‐pollinated control prevented a conclusive assessment of self‐incompatibility. Because colour, age, scent, reward and reproductive state covaried, their individual contributions to visitation could not be distinguished. Floral colour change may therefore form part of a multimodal indicator of floral state, while any display function of retained red flowers remains hypothetical. This within‐tree case study documents canopy visitation patterns whose generality requires replication across individuals and populations.

## Graphical abstract

Flowers of the threatened canopy tree *Firmiana daweishanensis* change from yellow to red as they age. Observations of 16 branches within one adult crown showed that yellow flowers received more visits and greater cumulative visitation time per flower‐hour than red flowers. These associations coincided with changes in other floral traits, preventing isolation of colour effects, and their generality requires replication across trees and populations.

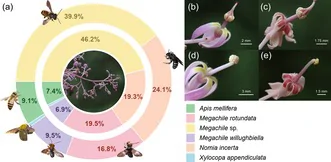

## 1 Introduction

Animal‐mediated pollination is a central ecological process shaping the reproduction of flowering plants. Floral traits, including colour, scent, morphology and nectar reward, influence how pollinators detect, approach and handle flowers, thereby affecting pollen transfer and reproductive success (Barrett and Harder 1996; Kunze and Gumbert 2001; van der Kooi et al. 2019). Among these traits, floral colour is one of the most conspicuous visual signals used by insects during foraging. It can influence both long‐distance attraction and fine‐scale choices among individual flowers (Bergamo et al. 2016; Garcia et al. 2020). However, floral signals are not always static. In some angiosperms, flowers change colour during anthesis, creating a dynamic display that changes as flowers age.

Floral colour change has long been interpreted as a potential cue that helps pollinators distinguish rewarding from older, less profitable flowers (Ruxton and Schaefer 2016; Weiss 1991, 1995; Weiss and Lamont 1997). Pollinators often prefer pre‐change flowers, whereas post‐change flowers may receive fewer visits. This pattern may reduce ineffective visitation and improve pollinator foraging efficiency (Ida and Kudo 2003; Ruxton and Schaefer 2016; Sun et al. 2005). Yet colour is unlikely to act alone. Floral scent and nectar reward can also shape pollinator learning and flower choice, and volatile compounds such as linalool are widely involved in plant‐insect communication (Knauer and Schiestl 2015; Kunze and Gumbert 2001; Zhang et al. 2023). Thus, floral colour change may represent more than a visual transition. It may function as a composite state signal in which visual conspicuousness, olfactory attraction, nectar reward and reproductive function change together.

Despite this framework, empirical studies of floral colour change have largely focused on herbaceous plants, shrubs, or other accessible systems. Direct behavioural evidence from tall rainforest canopy trees remains limited, partly because reproductive structures in the canopy are difficult to reach and manipulate. This gap is important because canopy trees contribute substantially to tropical forest structure and reproduction, yet their pollination ecology is often inferred indirectly. For rare canopy species, the problem is especially acute. When adult individuals are scarce, spatially isolated, or difficult for pollinators to connect, successful visitation may not translate into effective fertilisation (Kearns et al. 1998; Menz et al. 2011). Understanding whether floral signals still guide pollinator behaviour under these conditions is therefore relevant to both pollination ecology and the conservation of Plant Species with Extremely Small Populations.

*Firmiana daweishanensis* is a *Firmiana* species endemic to China and is listed as a National Class II protected wild plant (National Forestry and Grassland Administration 2021). It is also recognised as a Plant Species with Extremely Small Populations (PSESP), reflecting its restricted distribution, small population size and high conservation priority. As one of the few *Firmiana* species occurring in the limestone mountain rainforests of southern Yunnan, *F. daweishanensis* offers a rare system for examining how floral signals and pollinator behaviour interact in a threatened canopy tree. Understanding its pollination ecology is therefore important for interpreting the adaptive significance of floral colour change and for informing conservation of this narrowly distributed PSESP.

Here, we investigate whether floral colour stage in *F. daweishanensis* is associated with pollinator visitation in the rainforest canopy. We also examine accompanying variation in floral traits that may help explain this association. We first quantified the assemblage of effective hymenopteran pollinators visiting flowers across the accessible crown, including the total number of visits and cumulative visitation time recorded for each pollinator taxon. We then evaluated associations of floral colour, floral sex and their interaction with visit number and cumulative visitation time within a unified mixed‐model framework that accounted for flower availability and the grouping of observations within branches. We also examined whether branch‐level floral display, including the number and proportion of yellow and red florets, predicted visitation intensity.

To evaluate possible mechanisms, we measured linalool emission as an olfactory signal, nectar secretion as a floral reward, reflectance spectra and bee visual‐model contrasts as visual signals, and temporal changes in pollen viability and stigma receptivity as indicators of reproductive function. Finally, we compared pollinator exclusion, artificial geitonogamous self‐pollination and natural pollination to evaluate the contribution of floral visitors to fruit set and to obtain preliminary evidence concerning the breeding system. By integrating canopy visitation observations with floral trait measurements, this study examines whether the observed patterns are consistent with floral colour change forming part of a multimodal state signal.

## 2 Materials and Methods

### 2.1 Study Species and Floral Display

*Firmiana daweishanensis* is a Plant Species with Extremely Small Populations endemic to southeastern Yunnan, China. The species occurs in limestone rainforest valleys at low elevations and can grow into a tall canopy tree exceeding 30 m in height (Figure 1b). Its flowers are unisexual and borne in clustered panicles on flowering branches. Individual flowers change colour from yellow to orange and then red during anthesis.

**FIGURE 1:**
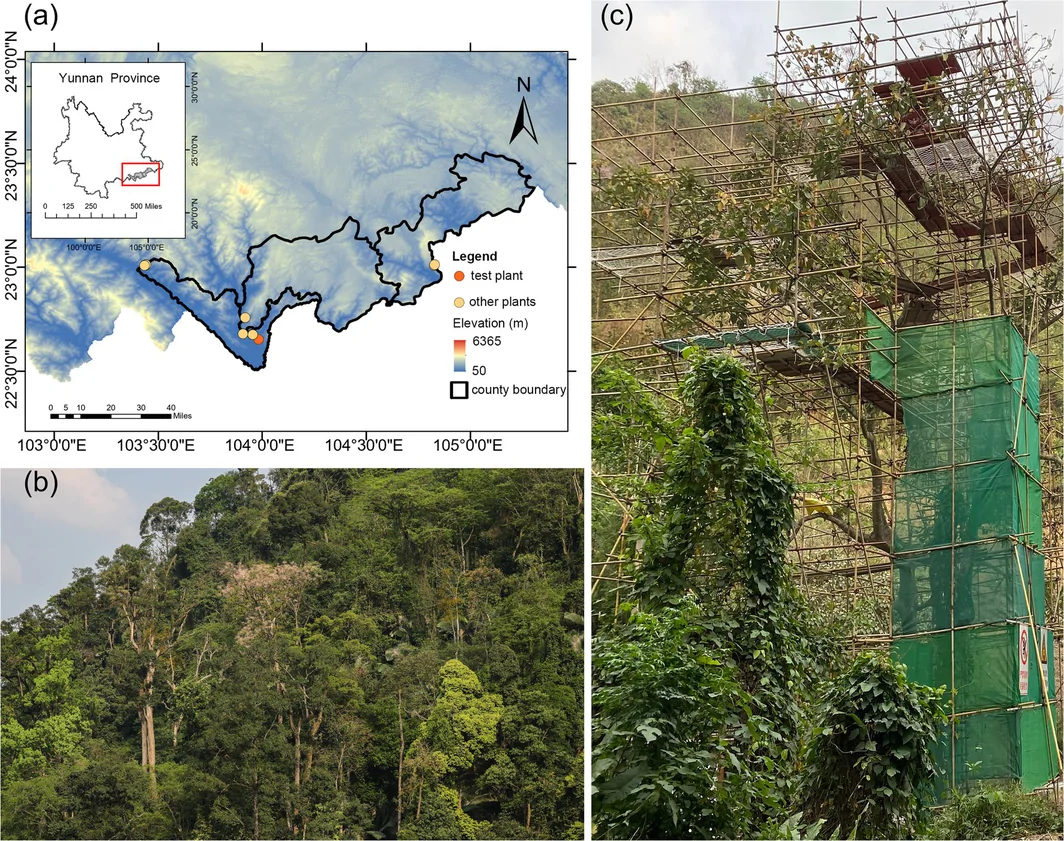
Study system and field access for *Firmiana daweishanensis*. (a) Distribution records and location of the study plant; (b) Limestone rainforest habitat of *F. daweishanensis*; (c) Canopy‐access scaffold used for pollinator observations. Photographs in panels (b) and (c) were taken by Bo‐Qiang Wang.

Field surveys were conducted across known distribution sites in Hekou County, Maguan County and Malipo County, Yunnan Province. Most adult individuals were located in dense limestone rainforest or on steep slopes and were not safely accessible for canopy observations. One accessible adult individual with a large crown was found near a roadside in Nanxi Town, Hekou County, Honghe Prefecture, Yunnan Province (22°39′ N, 103°58′ E; 224 m a.s.l.) (Figure 1a). A 25 m scaffold with five vertical levels was constructed around this tree, allowing access to different heights and directions of the crown (Figure 1c). All pollinator observations and floral trait measurements for this study were conducted on or around this accessible individual during March and April 2024.

Because inflorescences of *F. daweishanensis* are clustered on flowering branches, flowering branches were used as the main observational unit for pollinator behaviour. For each observed branch, all visible inflorescences were recorded. The numbers of female flowers, male flowers, yellow flowers, and red flowers were counted for each inflorescence. These counts were used to calculate flower abundance and colour composition at the branch level.

### 2.2 Pollinator Assemblage and Canopy Visitation Observations

Pollinator observations were conducted from the scaffold within the accessible crown. Preliminary observations recorded diverse flower visitors, including insects from several orders. Formal analyses focused on six hymenopteran insect species that showed stable and effective flower‐visiting behaviour. A visitor was considered an effective pollinator when it repeatedly contacted the reproductive structures of flowers during visits. To characterise the pollinator assemblage of the whole accessible crown, the total number of visits and cumulative visitation time were summarised for each of the six effective hymenopteran pollinators. These data were used to identify the dominant pollinator group.

For behavioural observations, 16 flowering branches were selected across the accessible crown. Each branch was observed from 9:00 to 17:00 for 8 h, giving a total of 128 branch‐hours of observation. During each observation period, we recorded pollinator identity, visit number, cumulative visitation time and the type of floret visited. Floret types were classified by colour and sex as yellow female, yellow male, red female and red male flowers. A visit was defined as the period from first contact with a flower until the visitor left that flower without immediately returning. Cumulative visitation time was measured in seconds.

### 2.3 Floret‐Level Visitation and Branch‐Level Display Effects

To quantify variation in pollinator visitation among floral types, observations were summarised separately for each flowering branch and each combination of floral colour (yellow or red) and floral sex (female or male). For each branch × colour × sex combination, we recorded the number of available flowers, total number of visits and cumulative visitation time. Observation exposure was expressed as flower‐hours and calculated as the number of available flowers multiplied by the 8‐h observation period. Visits per flower per hour and cumulative visitation time per flower per hour were calculated for descriptive summaries and visualisation, whereas the inferential analyses used the corresponding unstandardized response variables with flower‐hours included as an offset.

Floral colour remained approximately stable during the daytime observation period; the transition towards orange generally began around 18:00, with flowers becoming red by the following day. Flower numbers within the four colour–sex categories were therefore treated as constant during each 8‐h observation period, although occasional flower loss was not incorporated into the exposure calculation. Of the 64 possible branch × colour × sex combinations, two had no available flowers and were excluded because their exposure was zero. The analyses therefore included 62 observations from 16 branches: 15 observations for each female‐flower category and 16 for each male‐flower category. Six combinations with positive flower exposure but no visits were retained. These category‐level observations were grouped within branches rather than treated as independent replicates.

To examine associations between branch‐level floral display and pollinator visitation, we calculated five floral composition variables for each observed branch: yellow‐flower number, red‐flower number, the proportions of yellow and red flowers, and the ratio of yellow to red flowers. Spearman's rank correlations were used to examine the relationships between these branch‐level variables and pollinator visitation.

### 2.4 Floral Scent Collection and Analysis

Floral scent was sampled from four floral types: yellow female, yellow male, red female and red male flowers. Flowers were collected from the canopy and placed into 50 mL glass tubes sealed with silicone plugs. For each floral type, three replicate tubes were prepared, each containing approximately 30 flowers.

Volatile organic compounds (VOCs) were collected using solid‐phase microextraction with a 65 μm polydimethylsiloxane/divinylbenzene fibre (Supelco, USA). Ethyl caprate was added as an internal standard for quantification. Floral volatiles were analysed using gas chromatography–mass spectrometry (GCMS‐QP2020NX, Shimadzu, Japan) equipped with an Rtx‐5MS column. The ion source temperature was 230°C and the injector temperature was 260°C. The oven programme started at 40°C for 5 min, increased to 80°C at 20°C min^−1^ and held for 5 min, increased to 140°C at 5°C min^−1^, and finally increased to 260°C at 20°C min^−1^ and held for 2 min. Compounds were identified by comparison with the FFNSC1.3 and NIST libraries. Because linalool was the dominant floral volatile and is known to mediate plant‐insect communication (Zhang et al. 2023), subsequent analyses focused on linalool emission per flower.

### 2.5 Nectar Secretion

To quantify nectar reward during floral development, inflorescences were bagged before anthesis. Flowers that opened at 08:00 on the first day were marked for repeated nectar sampling. Nectar from male flowers was collected at 18:00 on the day of anthesis and at 04:00 and 14:00 on the following day. Nectar from female flowers was collected at the same times and additionally at 24:00 on the following day. Male flowers remained attached at the 14:00 sampling, when no nectar was recovered.

Nectar was collected using 5 μL capillary tubes. Thirty flowers of each sex were selected for repeated nectar sampling while they remained attached. Nectar volume was measured from the length of nectar in the capillary tube and expressed as microlitres per flower. The same flowers were sampled repeatedly at successive time points, and all available nectar was removed at each sampling. Subsequent measurements therefore represented nectar accumulated since the preceding collection.

### 2.6 Floral Reflectance Spectra and Bee Visual Modelling

To evaluate changes in visual signalling across floral development, reflectance spectra were measured at the yellow, orange, and red floral stages. Reflectance spectra were obtained from five different flowers at each colour stage, with each flower contributing one spectrum to the analysis (*n* = 5 flowers per stage). The colour‐changing regions of the calyx lobes were excised and arranged for measurement using a Maya 2000 Pro spectrometer coupled with a DH‐2000 light source (Ocean Optics, USA).

Reflectance data between 300 and 700 nm were interpolated at 1‐nm intervals and processed using the pavo package version 2.9.0 in R version 4.6.0. Negative reflectance values were corrected using the addmin method, and spectra were smoothed with a span of 0.15. Each replicate spectrum was processed and modelled separately.

Honeybee photoreceptor excitation was calculated using the vismodel() function in the pavo package with the following settings: visual = “apis”, illum = “D65”, trans = “ideal”, qcatch = “*E*_*i*_”, relative = FALSE, bkg = “green” and vonkries = TRUE. Receptor excitation was calculated as *E*_*i*_ = *Q*_*i*_/(*Q*_*i*_ + 1), producing excitation values for the ultraviolet‐, blue‐ and green‐sensitive photoreceptors (s, m and l, respectively).

Spectra were projected into the bee hexagon using the colspace() function in the pavo package with space = “hexagon”. Chromatic contrast against the adapted background was quantified as the Euclidean distance from the hexagon origin: $D_{\mathrm{hex}}=\sqrt{x^{2}+y^{2}}$, which corresponds to the r.vec output of colspace(). The origin represents the adapted green background. Green‐receptor achromatic contrast was calculated from the l output of vismodel(). Because the adapted background has an excitation value of 0.5 under the (*E*_*i*_) transformation, achromatic contrast was calculated as: *C*_G_ = |*E*_G_ − 0.5|. Stage‐mean spectra were used to display the reflectance curves and representative positions in Figure 5a,b. Replicate‐level (*C*_G_) values were used for the comparison between yellow and red floral stages in Figure 5c.

### 2.7 Reproductive Function and Pollination Treatments

Pollen viability, stigma receptivity and fruit set were measured to characterise reproductive function and determine whether self‐pollen could produce fruit under our experimental conditions. Pollen viability and stigma receptivity were assessed every 2 h from anthesis until flower wilting. Pollen viability was determined using in vitro germination. Preliminary tests identified a culture medium containing 30% sucrose and 0.003% boric acid, with 30 min incubation, as suitable for pollen germination. At each sampling time, pollen grains were incubated and examined under a microscope. The total number of pollen grains and the number of germinated pollen grains were counted, and pollen viability was calculated as the proportion of germinated pollen. Stigma receptivity was assessed using the benzidine‐hydrogen peroxide method following Dafni (1992). Stigmas were collected at the same time intervals as pollen samples, immersed in the test solution and scored according to reaction intensity.

To evaluate the contribution of floral visitors to fruit set and obtain preliminary evidence concerning the breeding system of *F. daweishanensis*, three pollination treatments were conducted. For the pollinator‐exclusion treatment, 100 female flowers were bagged before anthesis and left unmanipulated throughout anthesis, thereby preventing access by floral visitors. Qualitative observations were also made of the colour sequence in bagged, unmanipulated female flowers. The timing and rate of colour change were not quantified or compared statistically among pollination treatments. For artificial geitonogamous self‐pollination, 100 female flowers were bagged before anthesis, hand‐pollinated with pollen collected from male flowers on the same individual, and then rebagged to prevent external pollen deposition. For the natural‐pollination treatment, 872 female flowers were left exposed to floral visitors without manipulation. Fruit set was recorded for all three treatments.

### 2.8 Statistical Analyses

All statistical analyses were performed in R version 4.6.0. Pollinator visitation was analysed using generalised linear mixed models fitted with glmmTMB. The response variables were the total number of visits and cumulative visitation time in seconds for each branch × colour × sex combination. Visit counts were fitted using a negative‐binomial model with a quadratic mean–variance relationship (NB2) and a log link. Cumulative visitation time, which included zeros and positive continuous values, was fitted using a Tweedie model with a log link. Both models included floral colour, floral sex and their interaction as fixed effects, flowering branch as a random intercept, and the logarithm of flower‐hours as an offset. Flower‐hours were calculated as the number of available flowers multiplied by the 8‐h observation period. The random intercept accounted for the grouping of floral categories within branches; these observations were not analysed as independent, unpaired samples.

The full colour × sex models were retained for all primary analyses, irrespective of interaction significance. Likelihood‐ratio tests compared each full model with three reduced models. The overall contribution of colour was tested by jointly removing colour and the colour × sex interaction, leaving a model containing sex only; the overall contribution of sex was tested by jointly removing sex and the interaction, leaving a model containing colour only. Each omnibus test therefore had two degrees of freedom. The interaction was tested by comparing the full model with the corresponding additive model, giving one degree of freedom. Holm correction was applied jointly to the six primary tests across the two response variables.

Secondary comparisons were estimated from the full models using emmeans. These comprised yellow‐to‐red contrasts within each sex and male‐to‐female contrasts within each colour, giving eight comparisons across the two response variables. Their Wald‐test *p* values were adjusted together using the Holm method. Response‐scale ratios are reported with pointwise 95% Wald confidence intervals, which were not adjusted for simultaneous coverage. Model‐estimated rates for visualisation were calculated for one flower‐hour with the branch random intercept set to zero.

Model convergence and Hessian status were checked, and simulation‐based residual diagnostics were conducted using DHARMa, including tests of uniformity, dispersion, zero inflation and outliers. To assess the assumption that expected visitation increased proportionally with flower number, sensitivity models replaced the flower‐number component of the offset with an estimated coefficient for log flower number, while retaining log observation hours as an offset. These models retained colour, sex, their interaction and the branch random intercept. The estimated flower‐number coefficient was compared with one using a likelihood‐ratio test, and the colour, sex and interaction tests were repeated. Additional sensitivity analyses refitted the full models after omitting each branch in turn.

Spearman's rank correlations were used to test the relationships between branch‐level floral composition and pollinator visitation. All behavioural observations were obtained from 16 branches of one adult tree on one day. Branches therefore provided within‐crown observational replication, not independent replication among trees or dates. Accordingly, inference was restricted to associations within the observed crown during the observation period. Floral scent emission, nectar secretion, pollen viability and stigma receptivity were summarised as means with standard errors unless otherwise stated. Yellow‐ and red‐stage nectar volumes were compared separately for female and male flowers using two‐sided Wilcoxon signed‐rank tests, with measurements paired by flower identity. Yellow‐stage measurements represented accumulation from 08:00 to 18:00 on the first day, whereas red‐stage measurements represented accumulation from 04:00 to 14:00 on the following day, after complete nectar removal at 04:00. Both measurements therefore represented 10‐h accumulation periods. Green‐receptor achromatic contrast was compared between the yellow and red floral stages using Welch's two‐sample *t*‐test, with individual spectra treated as the statistical units (*n* = 5 per stage). Fruit set was summarised descriptively for each pollination treatment; no inferential comparison between treatments was conducted.

## 3 Results

### 3.1 Floral Colour Change and Pollinator Assemblage

*F. daweishanensis* flowered in the canopy of limestone rainforest, producing clustered inflorescences on exposed flowering branches. Both female and male flowers showed a conspicuous colour transition during anthesis, changing from yellow to orange and then to red before senescence (Figure 2b–e). Thus, yellow and red flowers co‐occurred within the same flowering crown and often within the same flowering branches. Bagged, unmanipulated female flowers underwent the same yellow‐to‐orange‐to‐red sequence as naturally exposed flowers, with no apparent difference in the timing or rate of colour change based on qualitative observations.

**FIGURE 2:**
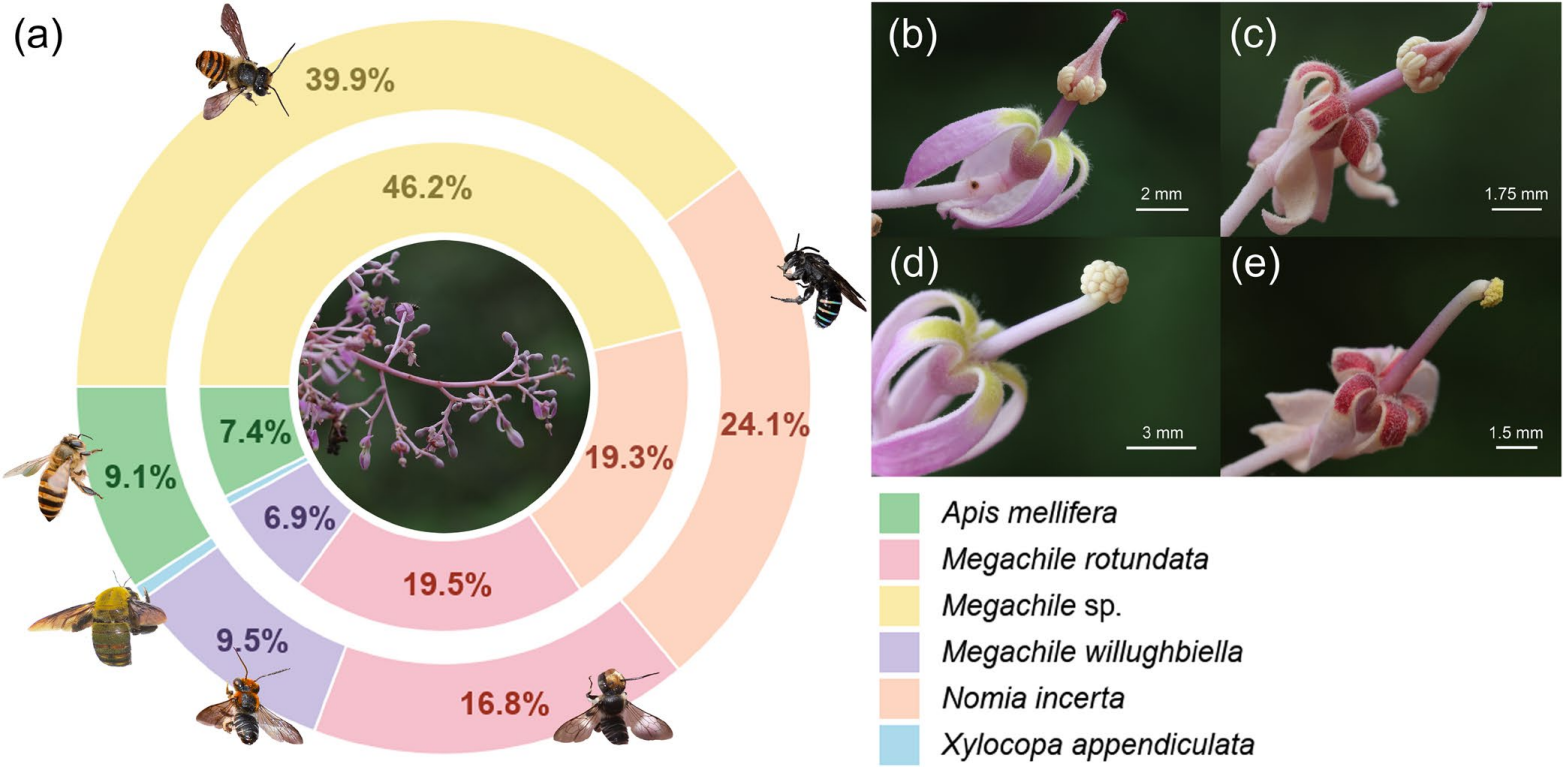
Floral colour change and pollinator assemblage of *Firmiana daweishanensis*. (a) Proportional contribution of six hymenopteran pollinators to total visit number and total cumulative visitation time. The inner ring indicates the proportion of total visits, and the outer ring indicates the proportion of total cumulative visitation time. (b) Yellow female flower. (c) Red female flower. (d) Yellow male flower. (e) Red male flower. All photographs were taken by Bo‐Qiang Wang.

Across whole‐plant observations, six hymenopteran pollinators were recorded visiting the flowers. The pollinator assemblage was dominated by leafcutter bees. *Megachile* sp. accounted for the largest proportions of pollinator activity, representing 46.2% of all visits and 39.9% of cumulative visitation time (Figure 2a). Together, the three *Megachile* categories accounted for most pollinator activity, whereas *Nomia incerta* , *Apis mellifera* , and *Xylocopa appendiculata* contributed smaller proportions (Figure 2a).

### 3.2 Pollinator Visitation Was Associated With Floral Colour Stage

Within the observed tree, pollinator visitation at the floret level was strongly associated with floral colour stage. Yellow flowers received 0.170 ± 0.225 visits flower^−1^ h^−1^, compared with 0.022 ± 0.020 visits flower^−1^ h^−1^ for red flowers (mean ± SD). Cumulative visitation time was also greater for yellow flowers (0.631 ± 0.598 s flower^−1^ h^−1^) than for red flowers (0.116 ± 0.121 s flower^−1^ h^−1^). These descriptive values are means ± SD of branch‐level rates calculated separately for each colour after pooling female and male flowers within branches (*n* = 16 branches).

After accounting for flower‐hours and the grouping of observations within branches, the omnibus colour tests were significant for both visit number (likelihood‐ratio test: *χ* ^2^ = 29.057, df = 2, Holm‐adjusted *p* = 2.45 × 10^−6^) and cumulative visitation time (*χ* ^2^ = 30.920, df = 2, Holm‐adjusted *p* = 1.16 × 10^−6^; Figure 3; Table S1). The omnibus sex tests were not significant for visit number (*χ* ^2^ = 1.934, df = 2, Holm‐adjusted *p* = 1.000) or cumulative visitation time (*χ* ^2^ = 3.963, df = 2, Holm‐adjusted *p* = 0.551). Neither colour × sex interaction was significant (visit number: *χ* ^2^ = 0.325, df = 1; cumulative visitation time: *χ* ^2^ = 0.443, df = 1; both Holm‐adjusted *p* = 1.000).

**FIGURE 3:**
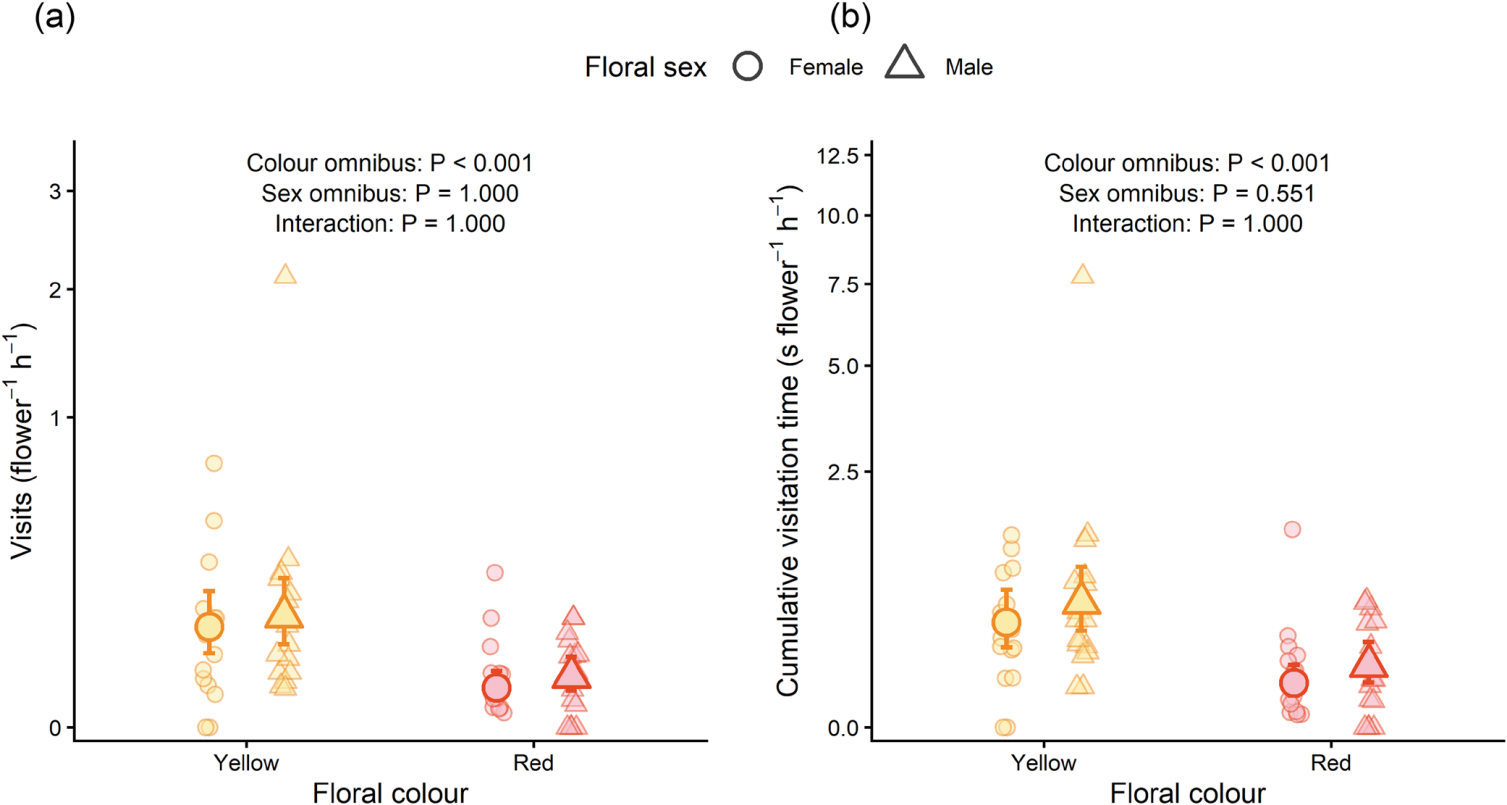
Pollinator visitation in relation to floral colour and sex within the observed crown. (a) Visits per flower per hour. (b) Cumulative visitation time per flower per hour. Small symbols represent observed rates for individual branch × colour × sex combinations; large symbols and error bars show estimates and pointwise 95% confidence intervals from the full colour × sex mixed models. Circles denote female flowers and triangles denote male flowers. Visit counts were fitted using an NB2 model and cumulative visitation time using a Tweedie model, both with log links, a branch random intercept and log flower‐hours as an offset. Predictions were calculated for one flower‐hour with the branch random intercept set to zero. Each female‐flower category was represented on 15 branches and each male‐flower category on 16 branches, giving 62 observations from 16 branches observed on one tree on one day. Vertical axes use square‐root spacing, with labels shown in the original units. Displayed *p* values are Holm‐adjusted across the six primary likelihood‐ratio tests; colour and sex omnibus tests have two degrees of freedom and interaction tests have one degree of freedom (Table S1).

Secondary comparisons from the full models indicated higher visitation to yellow than to red flowers within both sexes. Yellow‐to‐red ratios for visit rates were 6.40 (95% CI: 3.04–13.47) for female flowers and 4.80 (2.48–9.28) for male flowers. Corresponding ratios for cumulative visitation time per flower‐hour were 5.56 (2.84–10.86) and 4.06 (2.17–7.60), respectively. All four colour contrasts remained significant after Holm correction across the eight secondary comparisons (all adjusted *p* < 0.001). None of the within‐colour sex contrasts was significant after adjustment (Table S2).

Sensitivity analyses indicated that proportional scaling with flower number was imperfect. Estimated coefficients for log flower number were 0.710 (95% CI: 0.422–0.997) for visit number and 0.657 (0.388–0.926) for cumulative visitation time. Likelihood‐ratio tests against a coefficient of one gave unadjusted *p* values of 0.0499 and 0.0124, respectively. Nevertheless, the omnibus colour tests remained significant when flower‐number coefficients were freely estimated (both Holm‐adjusted *p* < 0.001). The colour association also remained detectable in all leave‐one‐branch‐out analyses (all unadjusted omnibus *p* < 0.001). Thus, the qualitative association with floral colour was robust to these checks, although the fixed‐offset model estimates should be interpreted in light of the proportional‐exposure assumption.

At the flowering‐branch level, neither the abundance nor the relative representation of yellow and red flowers was significantly associated with pollinator activity. Yellow‐flower number, red‐flower number, the proportions of yellow and red flowers, and the yellow‐to‐red flower ratio were not significantly correlated with either total visit number or cumulative visitation time (all *p* > 0.05; Figure S1). Floral colour stage was associated with visitation at the floret level, whereas no significant branch‐level correlations were detected. However, the sample of 16 branches may have limited our ability to detect such relationships.

### 3.3 Floral Signals, Rewards and Reproductive Function Changed Across Floral Stages

Yellow flowers showed stronger linalool emission than red flowers in both sexes (Figure 4a). Among the four flower types, yellow male flowers had the highest per‐flower linalool emission, whereas red male flowers had the lowest emission (Figure 4a). Yellow female flowers also emitted more linalool than red female flowers (Figure 4a).

**FIGURE 4:**
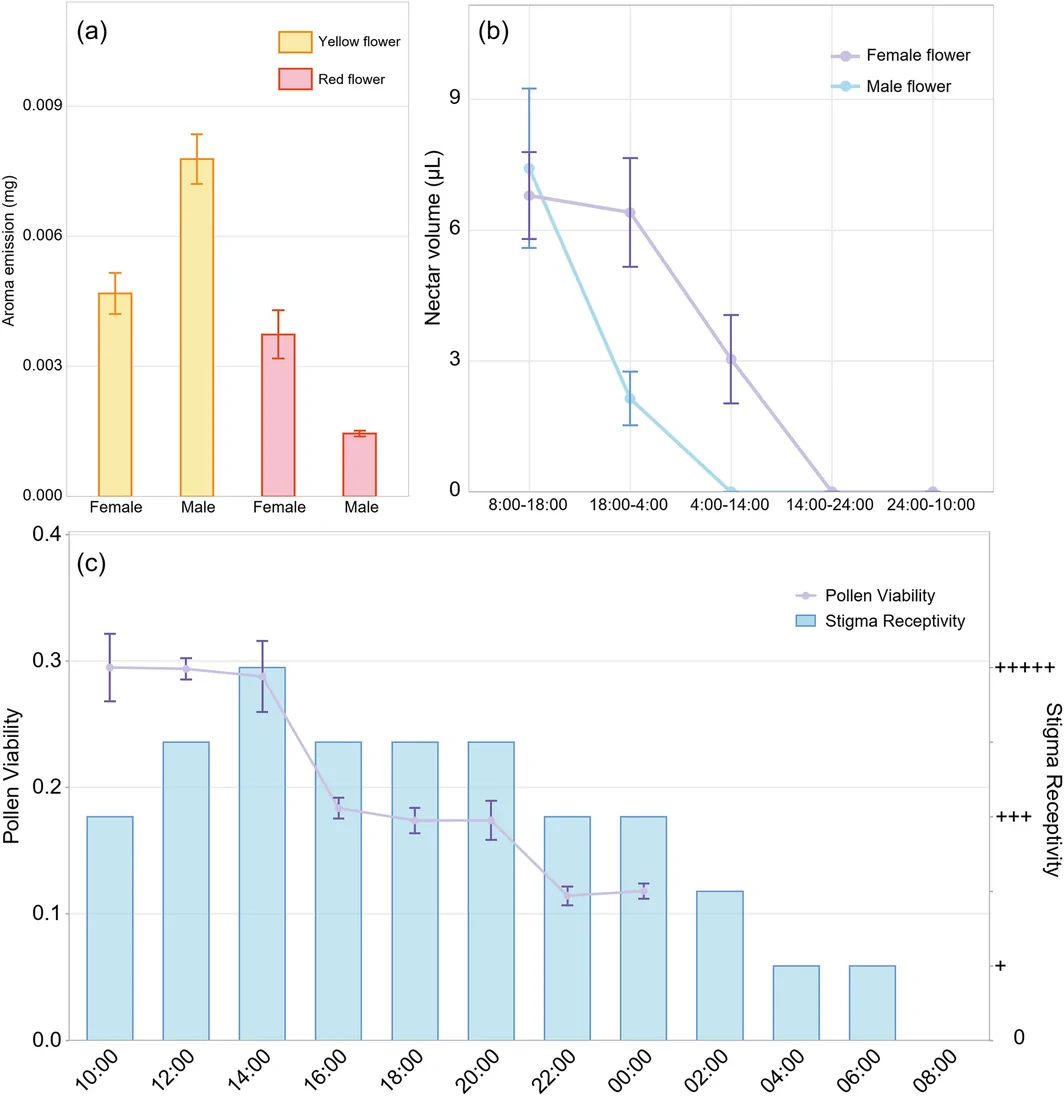
Floral scent, nectar reward and reproductive function during floral ageing. (a) Per‐flower linalool emission from yellow female, yellow male, red female, and red male flowers. (b) Nectar volume of female and male flowers across successive stages from flower opening to senescence. (c) Temporal changes in pollen viability and stigma receptivity during the floral lifespan.

Nectar volume varied during floral ageing (Figure 4b). Both female and male flowers contained relatively high nectar volumes at the first sampling time. In male flowers, no nectar was recovered at 14:00 on the second day, although the flowers remained attached until approximately 18:00. In female flowers, nectar volume accumulated over 10 h was greater during the yellow stage (08:00–18:00 on the first day) than during the red stage (04:00–14:00 on the second day; Table S3).

Reproductive function also changed through the floral lifespan. Pollen viability was highest during the early flowering period and then declined progressively (Figure 4c). Stigma receptivity increased after flower opening, reached its highest level around the middle of the observation period, and then decreased towards the end of anthesis (Figure 4c). Thus, the yellow to early‐transition stages coincided with higher floral reward and stronger reproductive function.

Reflectance spectra differed among the yellow, orange, and red floral stages. Yellow‐stage flowers showed higher processed reflectance across much of the bee‐visible wavelength range than orange‐ and red‐stage flowers (Figure 5a). In the bee hexagon model, the stage‐mean spectra occupied different positions, with the yellow‐stage spectrum located farther from the adapted‐background origin than the red‐stage spectrum (Figure 5b).

**FIGURE 5:**
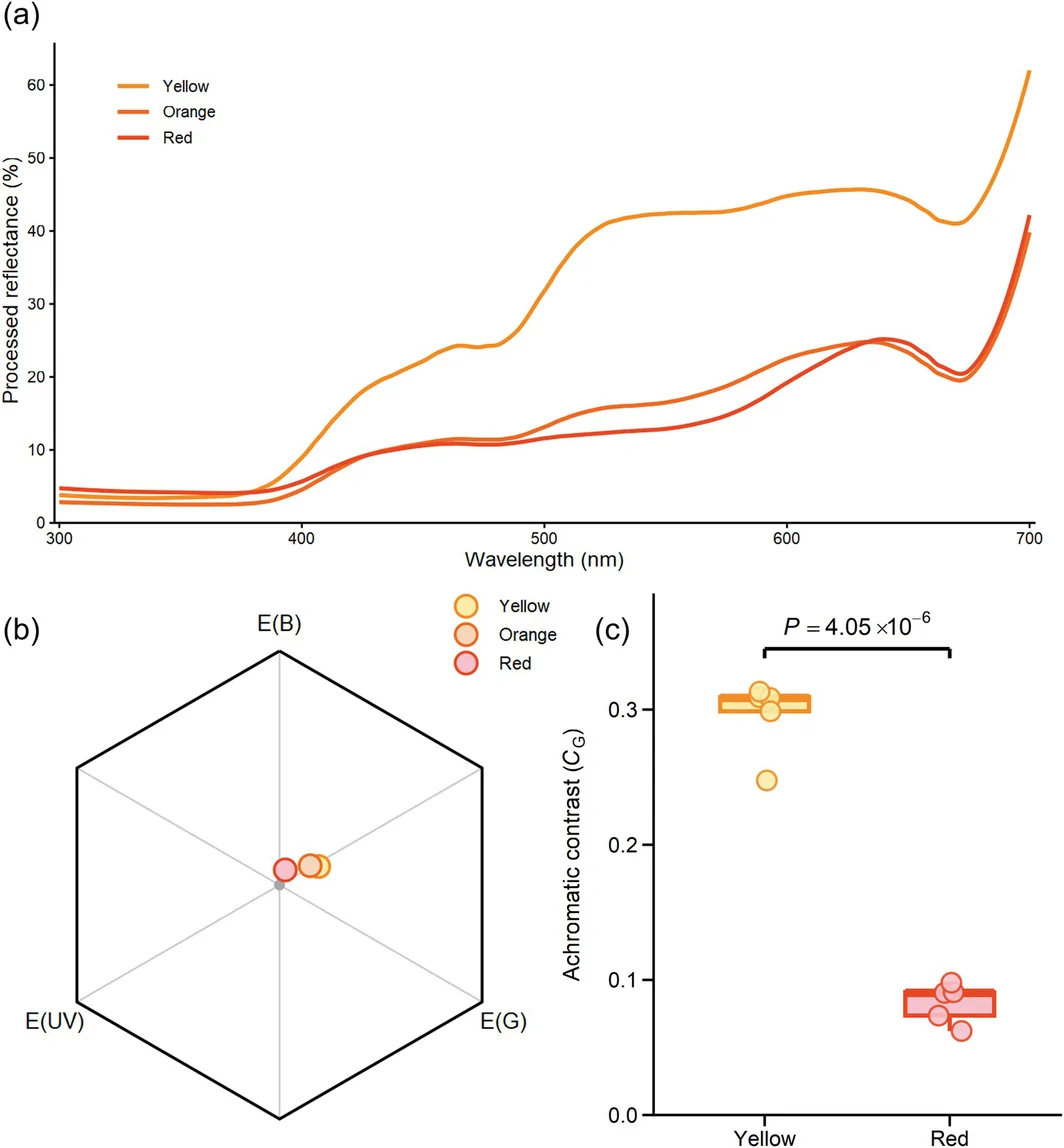
Reflectance spectra and bee visual modelling of floral colour stages. (a) Mean processed reflectance spectra of the yellow, orange and red floral stages, with each curve calculated from five replicate spectra. (b) Positions of the stage‐mean spectra in the bee hexagon colour space under D65 illumination and von Kries adaptation to the standard green background. Chromatic contrast against the adapted background was quantified as the Euclidean distance from the hexagon origin ($D_{\mathrm{hex}}=\sqrt{x^{2}+y^{2}}$). (c) Green‐receptor achromatic contrast, calculated as (*C*_G_ = |*E*_G_ − 0.5|), for individual yellow‐ and red‐stage spectra (*n* = 5 per stage). Points represent individual spectra; boxes show the median and interquartile range, and whiskers extend to 1.5 times the interquartile range. The displayed (*p*) value was obtained using Welch's two‐sample *t*‐test.

Green‐receptor achromatic contrast was significantly higher for yellow‐stage flowers than for red‐stage flowers (0.295 ± 0.027 vs. 0.083 ± 0.015, mean ± SD; Welch's two‐sample *t*‐test: (*t* = 15.326) (df = 6.130) (*p* = 4.05 × 10^−6^); Figure 5c). Thus, the transition from yellow to red was associated with a marked reduction in modelled green‐receptor contrast against the standard green background.

### 3.4 Fruit Set Differed Among Pollination Treatments

Neither pollinator‐excluded flowers (0/100) nor artificially geitonogamously self‐pollinated flowers (0/100) produced fruit. In contrast, naturally exposed flowers produced 54 fruits from 872 flowers, corresponding to a fruit set of 6.19% (Table 1). The absence of fruit under pollinator exclusion, together with fruit production under natural pollination, supports the contribution of floral visitors to fruit set under the conditions of this study. However, because no hand‐cross‐pollinated positive control was included, the breeding system and the presence of self‐incompatibility could not be determined conclusively.

**TABLE 1:** Fruit set under pollinator exclusion, artificial geitonogamous self‐pollination and natural pollination in *Firmiana daweishanensis*.

Treatment	Flowers	Fruits	Fruit set (%)
Pollinator exclusion	100	0	0
Artificial geitonogamous self‐pollination	100	0	0
Natural pollination	872	54	6.19

## 4 Discussion

Our within‐tree observations showed that floral colour stage was associated with pollinator visitation. Yellow flowers received more visits and greater cumulative visitation time per flower‐hour than red flowers within both sexes, after accounting for flower availability and the grouping of observations within branches. Neither the omnibus sex tests nor the colour × sex interactions were significant, although these results do not establish equivalent visitation between sexes or identical colour associations across sexes. The colour association persisted when the flower‐number coefficient was freely estimated and when individual branches were omitted. This pattern is consistent with the interpretation that floral colour change can form part of a signal of floral reward or reproductive state (Ruxton and Schaefer 2016; Weiss 1991). However, it does not demonstrate that colour itself caused the observed visitation pattern, because colour covaried with floral age, scent, nectar availability and reproductive function. Greater cumulative visitation time also does not necessarily imply longer individual visits. The contrast between floret‐ and branch‐level patterns is consistent with colour‐dependent selection operating at a relatively fine spatial scale, but does not establish the spatial scale of selection. With only 16 branches, nonsignificant branch‐level correlations may also reflect limited statistical power.

The partial nature of floral colour change and the retention of numerous red‐stage flowers raise the hypothesis that these flowers contribute to crown‐level display. In many colour‐changing species, older or less rewarding flowers are retained and may contribute to long‐distance floral display while younger flowers guide close‐range visitation (Ruxton and Schaefer 2016; Weiss 1995). In *F. daweishanensis*, the colour‐changing region is restricted mainly to the inner part of the calyx lobes. Much of the outer calyx lobe area remains pink throughout anthesis and fades only slightly as the flower ages. This stable outer region could continue to contribute to the visible floral display after the central region has changed from yellow to red. Accordingly, the flowering crown appears predominantly pink from a distance even when many individual flowers have red centres. Red‐stage flowers accounted for an average of 44.54% of the observed floral units. Their immediate abscission would therefore substantially reduce the number of flowers retained within the crown, although its effect on long‐distance attraction was not measured. We hypothesise that retaining red‐stage flowers may help preserve crown‐level display, whereas the yellow‐to‐red transition in the central calyx lobe region may provide a finer‐scale cue within the crown. However, we did not manipulate red‐flower retention, quantify display visibility or measure pollinator attraction from a distance. This proposed functional separation between crown‐level display and within‐crown signalling therefore requires experimental verification.

Several lines of evidence indicate that the preference for yellow flowers was associated with multiple floral traits rather than colour alone. Yellow flowers showed higher linalool emission than red flowers, and yellow male flowers had the highest emission among the four flower types. Floral scent is a major component of plant‐pollinator communication, and both the identity and emission rate of volatile compounds can influence pollinator attraction, learning and flower constancy (Knudsen et al. 2006; Raguso 2008; Schiestl 2010). In *F. daweishanensis*, stronger linalool emission in yellow flowers may therefore reinforce colour‐based discrimination, particularly for bees that integrate visual and olfactory information during foraging. Reward availability also varied during floral ageing. Female flowers accumulated more nectar during the yellow‐stage 10‐h interval than during the red‐stage interval. In male flowers, no nectar was recovered at 14:00 on the second day, although the flowers remained attached. These observations describe changes in recoverable nectar during floral ageing; the stage comparisons also involved different periods of the day. At the same time, pollen viability and stigma receptivity were higher during earlier floral stages and declined later in anthesis. These patterns indicate that yellow or early‐transition flowers were more closely aligned with both pollinator reward and reproductive function. The colour change from yellow to red therefore appears consistent with an honest signal of declining floral reward or reproductive value, a mechanism proposed for many colour‐changing flowers (Ruxton and Schaefer 2016; Weiss 1995). However, scent, nectar and spectral traits were measured on flowers or branches separate from those used for pollinator observations. These complementary observations suggest possible explanations for the visitation pattern but do not directly test the underlying mechanisms. The bee visual model was consistent with the observed association between floral colour and pollinator visitation. Yellow‐stage spectra generated greater green‐receptor achromatic contrast than red‐stage spectra and were located farther from the adapted‐background origin in the bee hexagon. These outputs indicate that yellow‐stage flowers produced stronger modelled visual contrast under D65 illumination and adaptation to a standard green background. They should not, however, be interpreted as a direct measurement of flower detection under natural canopy conditions. The stronger modelled contrast of yellow‐stage flowers was consistent with their higher visitation rate and greater cumulative visitation time per flower per hour. Together with the scent and nectar results, this pattern suggests that yellow‐stage flowers present a coordinated combination of visual signals, floral scent and reward. However, because these traits were measured separately and were not manipulated experimentally, their individual contributions to pollinator choice cannot be distinguished.

Pollinator‐excluded flowers produced no fruit (0/100), whereas naturally exposed flowers achieved a fruit set of 6.19% (54/872). This contrast provides experimental support that access by floral visitors contributed to fruit production under our study conditions. Artificial geitonogamous self‐pollination also produced no fruit (0/100), showing that self‐pollen did not result in successful fruit set under the conditions of our experiment. For a Plant Species with Extremely Small Populations, failure to obtain fruit through self‐pollen could increase reproductive vulnerability if compatible pollen donors are scarce, although cross‐compatibility was not tested here (Ashman et al. 2004; Kearns et al. 1998; Yang et al. 2022). However, because we did not include a hand‐cross‐pollinated positive control, this result cannot conclusively distinguish self‐incompatibility from other possible causes of self‐pollination failure. Controlled comparisons of self‐ and cross‐pollination using multiple individuals will therefore be required to establish the breeding system of *F. daweishanensis*.

Observations under pollinator exclusion suggest an age‐ or development‐related component to floral colour change. Bagged female flowers underwent the same yellow‐to‐orange‐to‐red sequence as naturally exposed flowers, with no apparent difference in transition timing or rate. These qualitative observations suggest that floral visitation is not required for the transition. However, without quantitative comparisons of transition timing, subtle effects of pollination cannot be excluded. A useful comparison is *Saxifraga fortunei*, in which pistils often change from yellow to red after pollination, with reddening associated with pollen‐tube progression towards the ovules (Takizawa et al. 2026). In *F. daweishanensis*, colour change occurs mainly in the inner region of the calyx lobes and proceeds under pollinator exclusion. This comparison highlights the need to distinguish developmental and pollination‐associated contributions to superficially similar floral colour changes.

These findings also point to practical conservation priorities. The observed fruit‐set pattern supports conserving floral visitors, flowering individuals and potential pollen donors, although the breeding system remains unresolved. These measures include protecting flowering individuals and nearby potential pollen donors, maintaining canopy and forest‐edge habitats used by bee pollinators, reducing pesticide or disturbance risks during flowering, and monitoring the activity of dominant pollinators such as *Megachile* bees. Where natural fruit set remains low, assisted outcross pollination, seed collection and ex situ propagation could be considered as complementary measures, but such actions should be based on compatibility tests and genetic information to avoid narrowing the genetic base of this PSESP. More broadly, this study adds a canopy perspective to floral colour‐change research. Tropical forest canopies are biologically rich but difficult to access, and pollination studies have historically been biased towards lower and more accessible vegetation (Bawa 1990; Lowman and Rinker 2004; Ozanne et al. 2003). By using a scaffold to observe flowers within the crown of a tall rainforest tree, our study documents an association between floral colour stage and fine‐scale pollinator visitation in situ in a canopy system. This is particularly relevant for *F. daweishanensis*, because most known individuals occur in steep, densely vegetated limestone rainforest where direct canopy observation is rarely possible. For Plant Species with Extremely Small Populations, understanding reproductive biology is a necessary step towards identifying causes of rarity and designing targeted conservation actions. Thus, the pollination biology of *F. daweishanensis* not only enriches our understanding of floral colour change in canopy trees, but also provides a biological basis for conserving this narrowly distributed and highly threatened species.

Several limitations remain. First, behavioural observations were conducted on one accessible adult tree, which reflects the logistical constraints of studying a Plant Species with Extremely Small Populations but limits population‐level inference. Observations across additional flowering individuals, dates and populations are needed to assess the generality of these patterns. Second, floral scent, nectar and reflectance were not measured on the same branches used for pollinator observations, because doing so would have disturbed natural visitation. Third, the observed patterns are consistent with a possible role for multiple floral traits, but they do not establish their individual or combined effects on pollinator visitation. Future work should combine manipulative experiments, such as colour alteration, scent addition or removal, and nectar supplementation, with bee visual modelling and pollinator choice assays. Such experiments would allow the relative contribution of visual signals, olfactory signals and reward status to be separated more directly.

Overall, floral colour stage was associated with pollinator visitation within the observed crown of *F. daweishanensis*. Yellow‐stage flowers received more visits and greater cumulative visitation time per flower‐hour than red‐stage flowers. Yellow‐stage flowers also exhibited stronger modelled visual contrast and higher linalool emission. These patterns are consistent with floral colour change forming part of a multimodal indicator of floral state, but do not establish which traits drove visitation. Retained red‐stage flowers may contribute to the crown‐level display, although this proposed function requires experimental verification.

## Author Contributions

**Bo‐Qiang Wang:** data curation (equal), formal analysis (lead), investigation (equal), methodology (lead), writing – original draft (lead), writing – review and editing (equal). **Si‐Yue Xiao:** investigation (supporting). **Cheng‐Jie Li:** investigation (supporting). **Yan‐Mei Ping:** investigation (supporting). **Jin‐Guo Zhang:** investigation (supporting). **Wei‐Bang Sun:** investigation (supporting), methodology (equal), project administration (equal). **Jing Yang:** formal analysis (equal), funding acquisition (lead), investigation (lead), methodology (lead), project administration (lead), writing – original draft (equal), writing – review and editing (lead).

## Funding

This study was financially supported by the Science and Technology Plan Project of Yunnan (grant no. 202201AS070049), the National Natural Science Foundation of China (grant no. 32371743), and the CAS Light of West China Program (to J.Y.).

## Conflicts of Interest

The authors declare no conflicts of interest.

## Supporting information


**Figure S1:** Relationships between branch‐level floral colour composition and pollinator visitation. Correlations between flowering‐branch traits and pollinator activity. Branch traits include yellow flower number, red flower number, yellow flower percentage, red flower percentage and the yellow:red flower ratio. Pollinator activity is represented by total visit number and total cumulative visitation time.
**Table S1:** Primary likelihood‐ratio tests from full colour × sex mixed models of pollinator visitation.
**Table S2:** Secondary within‐sex colour contrasts and within‐colour sex contrasts from the full mixed models.
**Table S3:** Nectar volume accumulated during the yellow and red floral stages.

## Data Availability

The data, metadata and R code supporting the findings of this study are available in the Figshare Digital Repository at https://figshare.com/s/32a63745b3051414b17e. The deposited files include the raw and processed data for pollinator visitation, branch‐level floral composition, floral scent, nectar volume, floral reflectance and bee visual modelling, pollen viability, stigma receptivity and fruit set, together with the source data underlying the figures and tables and the R scripts used for statistical analyses.
