# Supplementary material for: Pollinator Visitation Within a Single Crown of the Rare Rainforest Tree Firmiana daweishanensis Is Associated With Floral Colour Change
Source: Ecol Evol. 2026 Oct 12;16(10):e74490. doi: 10.1002/ece3.74490 (PMC13658850; doi:10.1002/ece3.74490)
Supplement: Supplementary file 1 [file ECE3-16-e74490-s001.docx]

**Appendix Tables and Figure**

**Table S1. Primary likelihood-ratio tests from full colour × sex mixed models of pollinator visitation.**

| **Response variable** | **Effect tested** | **χ²** | **df** | **Unadjusted P** | **Holm-adjusted P** |
| --- | --- | --- | --- | --- | --- |
| Number of visits | Colour omnibus | 29.057 | 2 | 4.902 × 10⁻⁷ | 2.451 × 10⁻⁶ |
|  | Sex omnibus | 1.934 | 2 | 0.380 | 1.000 |
|  | Colour × sex | 0.325 | 1 | 0.569 | 1.000 |
| Cumulative visitation time | Colour omnibus | 30.920 | 2 | 1.932 × 10⁻⁷ | 1.159 × 10⁻⁶ |
|  | Sex omnibus | 3.963 | 2 | 0.138 | 0.551 |
|  | Colour × sex | 0.443 | 1 | 0.506 | 1.000 |

Visit counts were analysed using an NB2 generalized linear mixed model and cumulative visitation time using a Tweedie generalized linear mixed model, both with log links. Full models included colour, sex, their interaction, a branch random intercept and log flower-hours as an offset. Colour and sex omnibus tests jointly removed the corresponding factor and its interaction (df = 2); interaction tests removed only the interaction (df = 1). Holm adjustment was applied across all six tests. Analyses included 62 observations grouped within 16 branches on one tree observed on one day.

**Table S2. Secondary within-sex colour contrasts and within-colour sex contrasts from the full mixed models.**

| **Response variable** | **Contrast** | **Ratio (95% CI)** | **Holm-adjusted P** |
| --- | --- | --- | --- |
| Number of visits | Yellow / red, female flowers | 6.40 (3.04–13.47) | 7.063 × 10⁻⁶ |
|  | Yellow / red, male flowers | 4.80 (2.48–9.28) | 1.889 × 10⁻⁵ |
|  | Male / female, red flowers | 1.64 (0.77–3.52) | 0.600 |
|  | Male / female, yellow flowers | 1.23 (0.66–2.31) | 0.600 |
| Cumulative visitation time | Yellow / red, female flowers | 5.56 (2.84–10.86) | 4.169 × 10⁻⁶ |
|  | Yellow / red, male flowers | 4.06 (2.17–7.60) | 5.713 × 10⁻⁵ |
|  | Male / female, red flowers | 1.93 (0.89–4.18) | 0.388 |
|  | Male / female, yellow flowers | 1.41 (0.83–2.38) | 0.600 |

Ratios compare model-estimated responses per flower-hour. All contrasts were estimated from the full colour × sex models. Wald-test P values were adjusted jointly across the eight comparisons using the Holm method. Confidence intervals are pointwise 95% Wald intervals and are not multiplicity-adjusted.

**Table S3. Nectar volume accumulated during the yellow and red floral stages.**

| **sex** | **yellow flower**  **(μL flower⁻¹ over 10 h)** | **red flower**  **(μL flower⁻¹ over 10 h)** | **Z** | **P** |
| --- | --- | --- | --- | --- |
| female | 6.793±0.993 | 3.045±1.009 | -2.714 | 7×10^-3^ |
| male | 7.417±1.827 | 0 | -5.442 | 5.2553×10^-8^ |

Values are means ± SE. Yellow- and red-stage nectar volumes represented accumulation from 08:00 to 18:00 on the first day and from 04:00 to 14:00 on the following day, respectively. Measurements were paired by flower identity and compared separately for each sex using two-sided Wilcoxon signed-rank tests. The zero value for red-stage male flowers represents an observed absence of recoverable nectar while the flowers remained attached.

**Figure S1. Relationships between branch-level floral colour composition and pollinator visitation.**

**Correlations between flowering-branch traits and pollinator activity. Branch traits include yellow flower number, red flower number, yellow flower percentage, red flower percentage and the yellow:red flower ratio. Pollinator activity is represented by total visit number and total cumulative visitation time.**
